# More support for Earth’s massive microbiome

**DOI:** 10.1186/s13062-020-00261-8

**Published:** 2020-03-04

**Authors:** Jay T. Lennon, Kenneth J. Locey

**Affiliations:** 1 Department of Biology, Indiana University, Bloomington, Indiana 47405 USA; 2 Rush University Medical Center, Chicago, Illinois 60612 USA

**Keywords:** Biodiversity, Scaling, Census, Biogeography, Macroecology

## Abstract

**Abstract:**

Until recently, our planet was thought to be home to ~ 10^7^ species, largely belonging to plants and animals. Despite being the most abundant organisms on Earth, the contribution of microbial life to global biodiversity has been greatly underestimated and, in some cases, completely overlooked. Using a compilation of data known as the Global Prokaryotic Census (GPC), it was recently claimed that there are ~ 10^6^ extant bacterial and archaeal taxa [1], an estimate that is orders of magnitude lower than predictions for global microbial biodiversity based on the lognormal model of biodiversity and diversity-abundance scaling laws [2]. Here, we resolve this discrepancy by 1) identifying violations of sampling theory, 2) correcting for the misuse of biodiversity theory, and 3) conducting a reanalysis of the GPC. By doing so, we uncovered greater support for diversity-abundance scaling laws and the lognormal model of biodiversity, which together predict that Earth is home to 10^12^ or more microbial taxa.

**Reviewers:**

This article was reviewed by Alvaro Sanchez and Sean M. Gibbons.

## Background

Over the last decade, enormous culture-independent inventories of microbial taxa have allowed biologists to address long-standing questions regarding the global diversity of microorganisms. Using the Global Prokaryotic Census (GPC), a collection of 16S rRNA gene sequences from 492 studies (34,368 sites), Louca et al. [1] concluded that Earth contains 0.8–1.6 million microbial taxa [1]. This estimate is six orders of magnitude lower than a prediction based on a comparably large data set of microbial communities [2]. Below, we demonstrate that the low estimate from [1] arises from violations of sampling theory and the misuse of biodiversity theory. After correcting for the misinterpretations of our previous work [2], we find that the GPC supports the prediction that there are at least 10^12^ microbial taxa on Earth.

Louca et al. [1] estimated microbial richness (i.e., number of taxa) at the global scale using approaches based on sampling theory that account for the frequencies of low-abundance classes (e.g., singletons, doubletons, etc.). These statistical estimators make no assumptions about biological processes and use more available information than approaches based on models of biodiversity [2]. However, such statistical estimators assume that unobserved taxa are present during sampling and that samples are unbiased representatives of the study system. Despite being one of the largest compilations of 16S rRNA gene sequences to date, the vast majority of samples in the GPC were obtained from central North America, central Europe, and Eastern China, while vast swaths of Earth are barely represented (see S1 Fig in [1]). Perhaps this geographical bias explains why 125,780 of the observed taxa (17%) were only recovered in one or two samples. Regardless, neither intuition nor evidence suggest the GPC is sufficiently representative of Earth’s microbiome to avoid the underestimation of global microbial diversity using statistical estimators. The authors did not acknowledge these violations of sampling theory, but instead concluded that “everything is everywhere” and then proceeded to use statistical richness estimators to predict global microbial diversity [1].

**Fig. 1:**
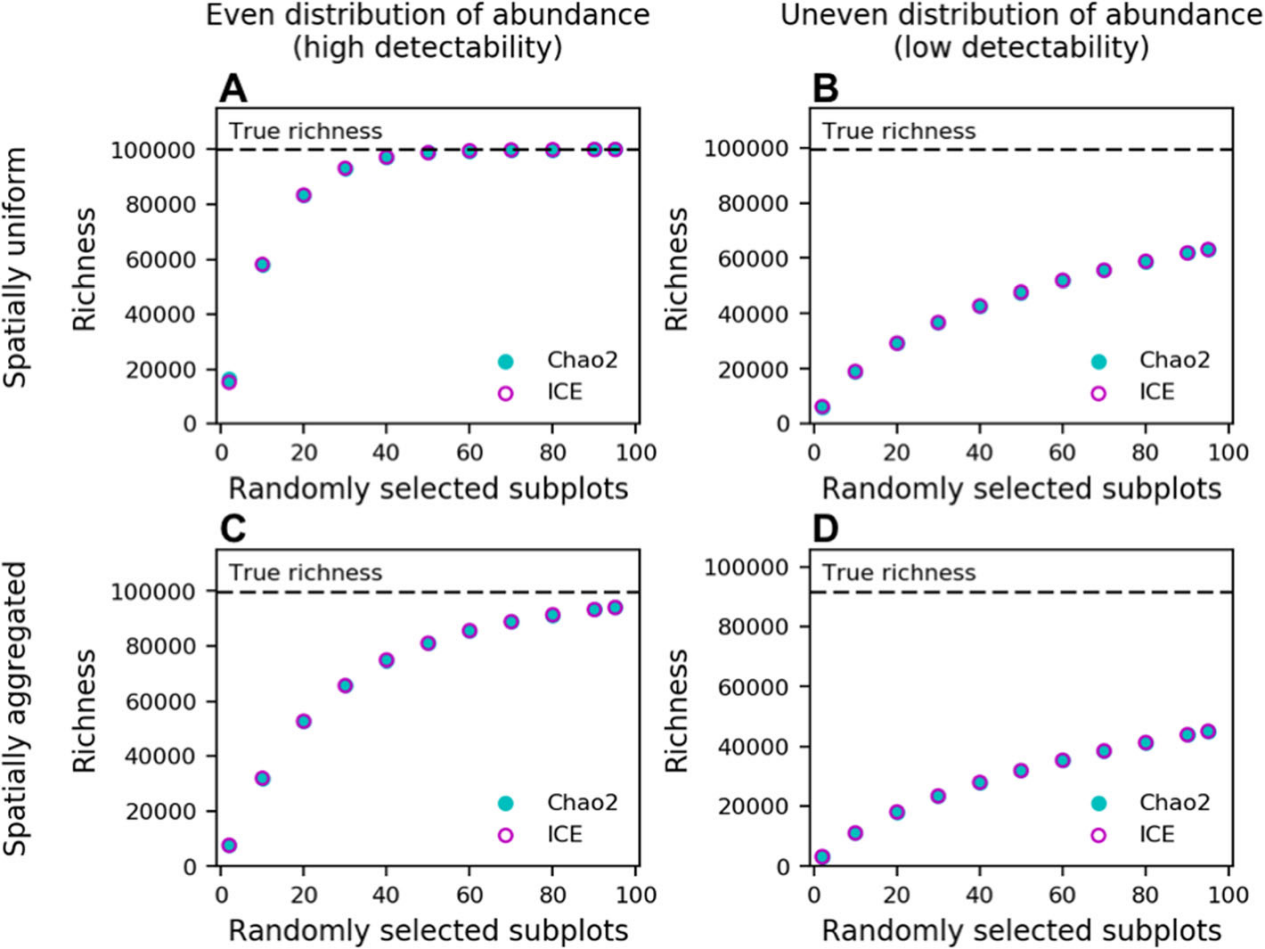
Results of distributing *N* = 10^7^ individuals belonging to *S* = 10^5^ species across a 2-D landscape (see S1 Fig). Subplots (**a**-**d**): Estimated and true richness under **a**) an unrealistic scenario where taxa are uniformly distributed in space with similar abundances, **b**) where species have uneven distributions of abundance but uniform distributions in space, **c**) where species have even distributions of abundance but aggregated distributions in space, and **d**) an ecologically realistic scenario where species have both uneven distributions of abundance and aggregated spatial distributions. For **c** and **d**, mean and standard deviations of normally distributed species spatial distributions where chosen at random. Information, source code, and simulated data can be found at https://www.github.com/LennonLab/census

To demonstrate how violations of sampling theory can affect richness estimation, we simulated the spatial distribution of 10^7^ individuals belonging to 10^5^ species under realistic and unrealistic scenarios. We randomly resampled increasing numbers of sites up to and including the entire simulated landscape before calculating richness using two common estimators (Chao2, ICE). Under an “everything is everywhere” scenario where taxa are similar in abundance and uniformly distributed in space, estimates quickly converged on the true richness of the system (Fig. 1a). Under these conditions, diversity estimators used by Louca et al. [1] and others [3] are justifiable and may perform better than other approaches that use less sample-based information, (e.g., [2]). However, when we simulated more realistic conditions where taxa have uneven abundances and are aggregated in space (see S1 Fig), richness was substantially underestimated even when all areas of the simulated landscape were sampled (Fig. 1b-d). Rather than explain the magnitude of discrepancy in real-world diversity estimates, our simulations simply illustrate why ecologists advise against using richness estimators when critical assumptions are violated [4] and why modern estimates for the global diversity of other taxa are hardly, if ever, based on such estimators.

**Fig. 2:**
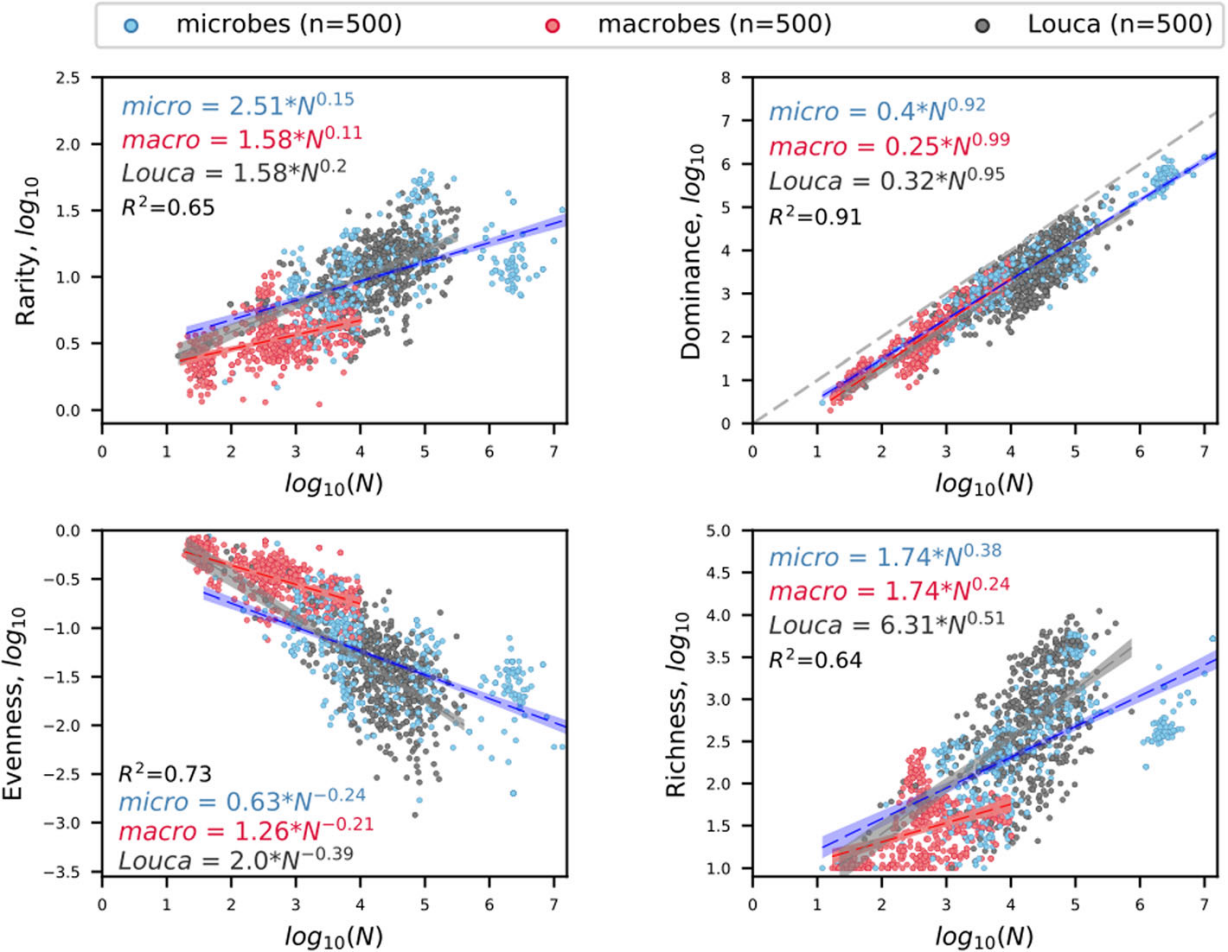
Diversity-abundance relationships (DARs) for microorganisms and macroorganisms (i.e., plants and animals) using data from [1, 2]. Abundance (*N*) refers to the number of individuals or sequence reads in a sample. Coefficients and exponents of scaling equations are mean values from 10,000 boot strapped multiple regressions, with each regression based on 500 assemblages chosen by stratified random sampling. Each scatter plot represents a single random sample; hulls are 95% prediction intervals. All code and data for recreating these analyses can be found at https://www.github.com/LennonLab/census

Aware of the limitations of richness estimators, we predicted global-scale microbial diversity using a combination of empirical scaling laws and a well-vetted model of biodiversity [2]. We began by documenting diversity-abundance relationships (DARs), which are statements of how rarity, dominance (*N*_*max*_), evenness, and richness scale with the total number of individuals or sequence reads (*N*). These DARs are not simply phenomenological but instead have been shown to emerge from interactions between biological processes, energetic constraints, and species traits [5]. Because Louca et al. [1] did not test whether the GPC exhibited DARs, we performed this task using their publicly available data. We found that the GPC supported DARs and that their scaling exponents were similar to those in our previous study (Fig. 2). For example, richness in the GPC scaled with abundance at a rate comparable (0.51 vs. 0.47) to that of the Earth Microbiome Project (EMP), which comprised > 70% of the microbial data in our study (Fig. S7-H in [2]). In addition, the GPC data supported the same nearly isometric scaling of *N*_*max*_ with *N* (*r*^2^ = 0.91), which held over 30 orders of magnitude [2].

In our original study, we based our formal predictions of richness for the human gut, cow rumen, global ocean, and all of Earth on the lognormal model of biodiversity and independent data obtained from previously published studies. The lognormal model had been rederived for predicting richness in large microbial systems [6] and requires only two empirical inputs: *N* and *N*_*max*_. Because the values of these inputs are at the same inherent scale as the value of the prediction, this approach is not, as others have incorrectly claimed (i.e., [1, 7]), an extrapolation. Instead, our approach simply assumes that 1) estimates of *N* and *N*_*max*_ from previous studies are reasonable and 2) that the global distribution of abundance among microbial taxa is lognormal. After arriving at a prediction of ~ 10^12^ microbial taxa on Earth, we then repeated the procedure using values of *N*_*max*_ that were predicted via the dominance DAR. In both cases, we found that the data supported an estimate of ~ 10^12^ species for Earth. In response to criticisms that were later raised [7], we reaffirmed the power of our approach by predicting global avian richness to within 6% of the modern estimate where, unlike microbes, the number of bird species is largely agreed upon [8]. A recent global analysis of bacteria from waste water treatment plants lends further support to our approach and the prediction of 10^12^ microbial taxa on Earth [9].

Despite claiming to refute our prediction of global microbial richness, Louca et al. [1] neglected to apply any of our approaches to their data. While they did use a lognormal model, they did so in an inappropriate way. Instead of using a lognormal model that takes global scale inputs (e.g., for *N* and *N*_*max*_) and returns global scale richness, they fit a lognormal species abundance distribution (SAD) to randomized aggregations of GPC data and then integrated across their fitted SAD to arrive at an estimate of ~ 10^6^ taxa. There are three critical problems with this approach. First, random combinations of data generate entirely artificial SADs. Regardless of whether the resulting SAD is lognormal or not, the result of this exercise is disconnected from the original non-randomized, non-aggregated data. Second, even if permitted, the results would only be pertinent to the data the model was fitted to, not the under-sampled biosphere. Regardless of how great a value of *N* was achieved through haphazardly combining sample abundances, the fitted model is irrelevant outside the context of the data it was fitted to. Third, integrating across a fitted SAD can hardly yield an estimate of richness that is orders of magnitude greater than the number of species observed in the data. Consequently, it is not surprising that estimated richness was in the same order of magnitude of what was observed [1].

After reanalyzing the GPC dataset using the appropriate lognormal approach [2, 6], we arrived at a prediction for global richness of ~ 10^14^ microbial taxa. In regard to orders of magnitude, this value is closer to the 10^12^ prediction of our previous study [2] than to the 10^6^ estimate from Louca et al. [1]. Consequently, the discrepancy between the census-based estimate of one million taxa [1] and the theoretically grounded prediction of one trillion taxa was not due to fundamental differences in the two comparably large data sets or even in the potential accuracy of the philosophically disparate approaches. Rather, the estimate that Earth’s microbiome is comprised of only 10^6^ taxa is the direct consequence of questionable assumptions and decisions that were made in the original analyses of the GPC data [1]. Given our findings and arguments in the current study, along with more recent estimates of massive microbiomes [9, 10] and the fact that immense regions of the planet remain unsampled, it is not beyond reason that Earth is home to 10^12^ microbial taxa or, at least, magnitudes more than 10^6^.

## Reviewers’ comments

### Reviewer 1, Sean M. gibbons, Institute for Systems Biology, University of Washington

Reviewer summary: The authors present a cogent piece that outlines how recent work that predicts only 10^6 bacterial species on Earth is analytically flawed (to me this 10^6 estimate seems, on its face, plainly wrong). They argue for why their scaling approach to estimating bacterial diversity is valid and how it provides an accurate prediction for global bird diversity (i.e. where the total # of species and # of individuals is relatively well known). They show simulation data that demonstrates how spatial heterogeneity (certainly lots of spatial heterogeneity in microbial ecosystems across the planet) can lead to underestimation of diversity when using the method from Louca et al. The piece is well-written and addresses an important topic.

Recommendation to authors: While I am sympathetic to the authors’ methods and conclusions, they may want to directly address the statistical criticism that came up previously (you address it indirectly by showing your method works for birds, but more exposition on ‘why’ your method works despite the critique would be useful). For example, Willis argues that the scaling approach is not statistically appropriate (https://www.pnas.org/content/113/35/E5096). The analogy that Willis provides in her piece (i.e. with bamboo, flies, pandas, and fish) is clear and compelling, but I believe that it makes incorrect assumptions from an eco/evo point of view. I think you should discuss this in another paragraph, where you explain that the process of sampling biological species is different from sampling colored marbles from a bag (unless those marbles are constantly speciating to create new colors). Bacterial species are constantly being generated over time, and this has been true for ~ 4 billion years. Species are lost over time too... Most models that takes these species birth/death processes into account will eventually give rise to scaling relationships between #individuals and # species. This is not simply some phenomenological artifact that lacks predictive power, but ultimately derives from mechanisms of evolution (diversification) and ecology (niche partitioning or spatial heterogeneity). The more cells that exist on Earth, the more rounds of cell division, the more mutations that occur, and the more chance for diversification/speciation (i.e. under certain conditions/assumptions, a correlation between # cells and # species should exist).

Authors’ response: *We directly addressed the concerns raised by citing two additional papers. In so doing, we point out the strengths of statistical estimators (see lines 63–65), but also highlight their limitations. As suggested, we also added a sentence (and reference) describing how our diversity-abundance scaling relationships emerge from interactions between biological processes, energetic constraints, and species traits (see lines 77–79).*

### Reviewer 2, Alvaro Sanchez, Department of Ecology and Evolutionary Biology, Yale University

Reviewer summary: In my opinion, the paper should be published, as it will contribute to an ongoing scientific conversation on a topic that seems far from resolved, and I believe that it makes several important points. I also want to emphasize that my review is focused on the paper under review, and that I do not intend to produce a critique of the original paper by Louca et al. Through my comments, I intend to suggest ways to improve the paper to increase its usefulness to the ongoing debate in the community. Summary: In this paper, Locey and Lennon respond to a recent paper by Louca and co-workers that provided an estimate of the number of prokaryotic OTUs of ~ 10^6, in contrast to prior studies that estimate this richness to be several orders of magnitude higher (10^12). The authors first briefly describe the study by Louca et al. These authors had analyzed a Global Prokaryotic Census (GPC) data base, which includes 16S sampling from ~ 500 studies in ~ 35,000 sites. The authors highlight in Fig. 1 some of the pitfalls associated to richness estimators, and in Fig. 2 they reanalyze the data from the GPC and plot various Diversity-Abundance Relationships, showing that the scaling relationships are consistent with the Earth Microbiome Project (EMP) data which had been data previously analyzed by Locey & Lennon. Finally, they apply to the GPC data the same procedures and Lognormal model they had previously used in their original paper, showing that the estimate number of taxa is ~ 10^14, not far from the ~ 10^12 estimate they had previously produced. Given the relative (very coarse grained) agreement between the two data sets when filtered through the same model, my impression is that, in the end, the matter rests on the validity of the lognormal model approach to extrapolate the total number of taxa on earth. The authors had previously shown that this model does an impressively good job at predicting the number of bird species on earth, a number that is well documented.

Recommendation to authors: Assessment: A fundamental challenge in this field is that the earth is a big planet with a number of microhabitats that vastly exceeds what we have been able to sample. Moreover, the large sizes of communities makes it challenging to sample the rarer members, a problem that is exacerbated by current technical limitations (e.g. the stringent filtering one needs to apply to remove sequencing errors, which often requires removing singletons, or the fact that only the V4 region is typically available). One is then forced to make inferences and extrapolations, which necessarily require assumptions. It is therefore not surprising that there are disagreements about these assumptions, and this has led to a lively field where contrasts of opinion are common (and I would say, healthy), and I strongly believe that this debate should be made public. I therefore support publication of this paper, as I believe it raises valuable points about assumptions in the interpretation of data, and it also provides a side by side reanalysis of the diversity-abundance scaling relationships of the GPC data in contrast with the Earth Microbiome Project data (which, encouragingly, appears to not be very different across both sets). My main suggestion would be that, at the end of the first paragraph, the authors state that they demonstrate that the estimate in the Louca et al. study arises from violations of sampling theory. My sense after reading the paper is that the authors do convincingly illustrate the point that, when taxa are not unevenly distributed and unevenly abundant, sampling approaches will underestimate abundance. Yet, Fig. 1 is not a direct attempt to estimate the potential effect of undersampling in the specific analysis of the Louca et al. study, so the statement could be toned down to more accurately reflect this point.

Authors’ response: *It is correct that our approach, which involves the diversity-abundance relationships (DARs) and the lognormal, is not intended to explicitly deal with the issue of undersampling. In our revision we clarify the advantages and disadvantages of this approach with that of statistical estimators while highlighting the consequences of violating assumptions for inference at the global scale. Based on reviewer feedback, we have attempted to make this distinction clearer with additional text (see lines 43–45, 63–65). Additionally, the paragraph starting on line 103 has been revised to more explicitly describe the use and misuse of the lognormal to estimate global richness.*

## Supplementary information

**Additional file 1.** Supplementary Information: More support for Earth’s massive microbiome
