# Supplementary material for: More support for Earth’s massive microbiome
Source: Biol Direct. 2020 Mar 4;15:5. doi: 10.1186/s13062-020-00261-8 (PMC7055056; doi:10.1186/s13062-020-00261-8)
Supplement: Supplementary file 1 — Additional file 1. Supplementary Information: More support for Earth’s massive microbiome [file 13062_2020_261_MOESM1_ESM.docx]

Supplementary Information: More support for Earth’s massive microbiome

Jay T. Lennon and Kenneth J. Locey

**
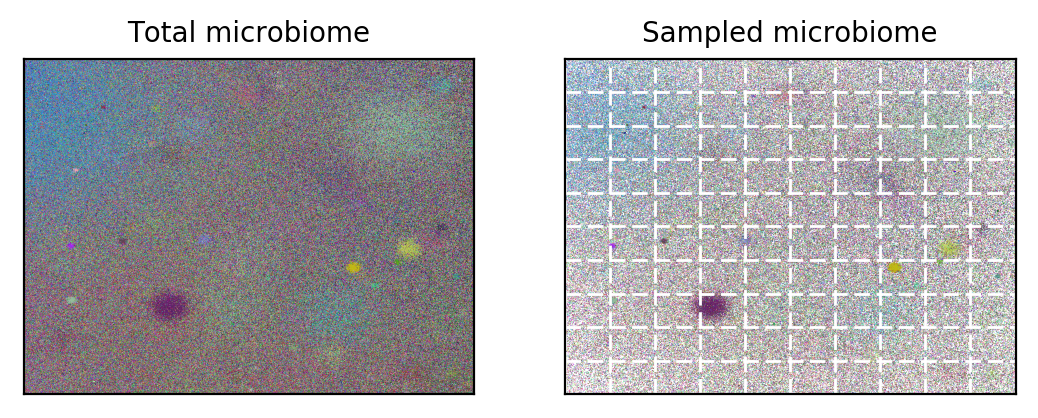
**

**S1 Fig.** Simulated 2-D landscape containing an abundant (10^7^ individuals) and diverse (10^5^ taxa) microbiome for assessing bias in richness estimators. Left panel depicts the unsampled microbiome where taxa have uneven distributions of abundance and are aggregated in space. Right panel represents the microbiome following random sampling. Source code and simulated data can be found at https://www.github.com/LennonLab/census.
